# Unmet needs in asthma treatment in a resource-limited setting: findings from the survey of adult asthma patients and their physicians in Nigeria

**DOI:** 10.11604/pamj.2013.16.20.2798

**Published:** 2013-09-18

**Authors:** Olufemi Olumuyiwa Desalu, Cajetan Chigozie Onyedum, Adekunle Olatayo Adeoti, Obianuju Beatrice Ozoh, Joseph Olusesan Fadare, Fatai Kunle Salawu, Ali Danburam, Ademola Emmanuel Fawibe, Olanisun Olufemi Adewole

**Affiliations:** 1 AffiliationDepartment of Medicine, University of Ilorin Teaching Hospital, Nigeria; 2 Department of Medicine University of Nigeria Teaching Hospital Ituku Ozalla Enugu, Nigeria; 3 Department of Medicine Ekiti State University Teaching Hospital Ado-Ekiti, Nigeria; 4 Department of Medicine, College of Medicine, University of Lagos, Lagos, Nigeria; 5 Department of Pharmacology, Ekiti State University Teaching Hospital Ado-Ekiti, Nigeria; 6 Department of Medicine, Federal Medical Centre Yola, Nigeria; 7 Department of Medicine, Obafemi Awolowo University Teaching Hospital Ile-Ife, Nigeria

**Keywords:** Asthma, education, communication, side effects, treatment compliance, treatment practices, barriers

## Abstract

**Introduction:**

The prevalence of asthma in our society is rising and there is need for better understanding of the asthma patients’ perception and treatment practice of physicians. The study was aimed at determining asthma attitudes and treatment practices among adult physicians and patients in Nigeria, with the goal of identifying barriers to optimal management.

**Methods:**

To assess asthma attitudes, treatment practices and limitations among adult physicians and patients in Nigeria, a questionnaire survey was conducted among 150 patients and 70 physicians.

**Results:**

Majority (66.7%) of the patients reported their asthma as moderate to severe, 42.7% had emergency room visit and 32% had admission due to asthma in the previous 12 months. Physicians and patients perceptions significantly differed in the time devoted to educational issues (31.4% vs.18.7%) and its contents: individual management plan (64.3% vs.33.3%), correct inhaler technique (84.0% vs.71.0%), medication side effects (80.0% vs.60.0%) and compliance 100% of time (5.7% vs. 18.7%). Patients reported that non-compliance with medication causes increased symptoms (67.0%), exacerbations (60.0%), bronchodilator use (56.0%), urgent physician visit (52.0%) and hospitalizations /ER visits (38.7%). Asthma medication in patients caused short term (10.7%) and long term side effects (20.0%). Due to side effects, 28.0% skipped and stopped their medications. Most physicians (85.7%) and patients (56.0%) agreed on the need for new medication options. The need for new medication in patients was strongly related to asthma severity, limitation of activities, side effects, cost and lack of satisfaction with current medication. With the exception of pulmonologists, physicians did not readily prescribe ICS and their prescriptions were not in line with treatment guidelines.

**Conclusion:**

This study has highlighted the gaps and barriers to asthma treatment which need to be addressed to improve the quality of care in Nigeria.

## Introduction

Asthma affects about 235 million people worldwide [1]. The incidence of asthma has been growing over the past 30 years due to changing environmental factors, particularly in the low- and middle-income countries that are least able to absorb its impact [1]. Asthma causes an estimated 250,000 deaths annually (1 in 250 deaths worldwide) [1, 2]. In addition, the World Health Organization estimates that around 15 million disability-adjusted life years (DALYs) are lost annually through this disease [2]. Fifty years ago asthma was uncommon in Nigeria, however recent reports from different parts of Nigeria have shown a prevalence of adolescent and adult asthma in excess of 10% and a rising trend in the prevalence of asthma [1, 3–7]. The increase in burden the asthma has been attributed to environmental factors such as urbanization, industrialization and adoption western life style [8]. The International Study of Asthma and Allergies in Childhood in children and the European Community Respiratory Health Survey in adults have greatly increased our understanding of epidemiology of asthma worldwide [9–11]. The Asthma Insights and Reality (AIR) surveys further gave more understanding into the actual variations in symptom severity and control of asthma and the current state of asthma management with respect to the GINA guidelines [12]. The AIR study found that significant proportion of patients continue to have symptoms, lifestyle restrictions and require emergency care. There is also a poor correlation between patients-perceived severity of asthma and objective assessment of severity on the basis of GINA criteria. The current level of asthma control worldwide falls far short of the goals for long-term management in international guidelines. [12]. Considering the report of AIR study which was attributed to gaps in the physician management and patient understanding of asthma causes and treatment, the Global Asthma Physician and Patient (GAPP) Survey [13] was designed to build on the findings from the AIR study, to uncover asthma attitudes and treatment practices among separate groups of physicians and patients, with the goal of identifying barriers to optimal management. In view of the rising prevalence of asthma in our society there is need for better understanding of the asthma patients’ perception and treatment practice of physicians. There is paucity of data on asthma attitudes and treatment practices among physicians and patients in Nigeria. The initial global survey excluded most developing countries and no similar study has been done in resource poor settings. Our study therefore was aimed at exploring the asthma attitudes and treatment practices among adult physicians and patients in Nigerian hospitals, with the goal of identifying barriers to optimal management.

## Methods

### Study design and population

This survey was a cross sectional study conducted from 30th March to 24th September, 2012. The study settings were six tertiary and three private (primary care) hospitals in five out of the six geopolitical regions of Nigeria. Nigeria is in the West African sub-region and it is the most populous nation in Africa. The GAPP study [13] protocol was adopted for this study and modified to suit our local setting. The modifications were in terms of administration of the survey instrument, sample recruitments and the types of health care providers recruited for the study, as nurses were not closely involved with treating Nigerian patients with asthma.

### Sample size

The minimum sample size was arrived at using Cochran's formula n = Z^2^pq/d^2^, n = Sample Size, p = prevalence of asthma among adults in Ilorin, Nigeria which is 15.2% [3]. The q = (1 - p), Z = standard normal deviation usually set at 1.96 which correspond to the 95% confidence interval. d = degree of accuracy desired usually set at 0.05. The calculated minimum sample size was 198. The population of adult asthma patients seen in the participating hospitals in the preceding one year before the study was 410. However, since this sample size exceeds 5% of the eligible population (400 x 5% = 20.0), Cochran's correction formula was used to calculate the final sample size. These calculations are as follows: n/ (1+n/410) = 134. Assuming a response rate of 90 %, a sample size of 147 was desired for adult patients. All eligible physicians working in participating hospitals and who met the inclusion criteria were recruited for the study.

### Patients and physicians selection

The inclusion criteria for patients were: asthma patients attending participating hospitals must be least 18 years of age and their clinical diagnosis of asthma made at least 6 months prior to the study. Patients with cognitive impairment, a severe exacerbation of asthma, or co-morbid chronic pulmonary disease (e.g., emphysema, chronic bronchitis, or bronchiectasis) were excluded from the study. For the physicians, the inclusion criteria to be eligible for recruitment were: working in the department of family and internal medicine, give written consent to participate in the study, practicing medicine for 3-30 years; sees at least three adult asthma patients per week; and writes at least one prescription for asthma medications per week. The investigators screened eligible patients and informed them about the study. The patients and physician who gave their consent and met the inclusion criteria were also recruited as study participants.

### Survey instrument

The questionnaire used in the study was a modification of the GAPP Survey questionnaire [13]. It was administered in English language, the official language in Nigeria. The questionnaire was pretested before use on 10 doctors and 10 patients in one study site to ensure the wording and content of the questions were widely understood and appropriate mode of administration of questionnaire was adopted. The questionnaire included items asking physicians and patients respondents to provide demographic information and answers to questions on the asthma diagnosis and symptoms, communication with their respective patients or physicians, resource utilization, experience with asthma medications, side effects from asthma medications, concern and awareness of side effects, treatment compliance and interest or desire for improved treatments. The patients and physicians were allowed to complete their questionnaire to ensure anonymity and guaranteed the confidential nature of the survey. Patients who had difficulty in completing the questionnaire were interviewed face-to-face by a trained interviewer who translated the items in the questionnaire into their native language. This mode of data collection was adopted to prevent exclusion of illiterate or patients with no formal education and to obtain the most representative sample from each participating hospitals.

### Data analysis

The questionnaires were reviewed manually for consistency and appropriate coding prior to data entry. The data were analyzed using the Statistical Package for the Social Sciences (SPSS), version 15 (SPSS Inc. Chicago IL, USA). Descriptive and frequency statistics were obtained for the variables of interest.

Chi square was used to test for statistical significance between categorical variables. Stratified analysis was performed to determine the relationship between impact of asthma, symptoms control, impact of medication side effect, level of satisfaction with current medication and the desire for new treatment options. All variables found to be significant were entered into bivariate analysis and Spearman's correlation coefficient was obtained. A P value of <0.05 was considered to be statistically significant.

## Results

We interviewed 150 patients and 70 eligible doctors comprising of family physicians (FP)/ general practitioners (GP) and specialists (i.e. Internist, pulmonologists). Of the 150 patients, 68% were females. General characteristics of the participating physicians and patients are shown in Table 1.

**Table 1:** Demographic profile of physicians and asthma patients

Profile	%
**Number of physicians**	70
**Specialty (%)**	
Pulmonology	8.6
Family medicine /GP	25.7
Internal medicine	55.7
Median amount of time in clinical practice (years)	8
**Mean no. of patients/week (%)**	
≤5	65.7
6-10	27.2
>10	7.1
**Asthma experience**	
Median no. of patients/week	4
Median no. of prescriptions/week	3.5
**Patients with asthma**	
**Number of patients**	150
**Gender**	
Male	32.0
Female	68.0
**Mean age (years)**	39(16)
**Time since diagnosis (years)**	
<5	41.3
5 to <10	21.3
10 to <15	17.3
15 to <20	4.0
20 to <30	9.3
30 +	6.7

### Impact of asthma and lack of symptom control

In this study, 33.3% of patients described their asthma severity as mild, 50.7% as moderate and 16.0% as severe. Almost 18.7% reported that asthma reduced their activity a great deal and 44.0% reported that they are somewhat limited by it. During the previous 12 months, 52.0% visited their physician for urgent care, 42.7% went to the hospital emergency room and 32.0% were admitted to hospital as a result of asthma (Table 2).

**Table 2:** Reported impact of asthma and lack of symptom control by patients (n=150)

Variables	%
**Severity of asthma**	
Mild	**33.3**
Moderate	**50.7**
Severe	**16.0**
**Limitation of activity**	
Not at all	**12.0**
Not much	**25.3**
somewhat	**44.0**
Great deal	**18.7**
**Health resource utilization**	
Unscheduled visit to doctor	**52.0**
Unscheduled telephone call	**22.7**
Visit Hospital ER	**42.7**
Admitted to Hospital	**32.0**

### Levels of asthma education

On their perception of asthma education, 38.6% of physicians and 18.7% of patients said that up to half of the clinic visit was devoted to educational issues. The physicians and patients perceptions significantly differed in 50% of clinic time devoted to educational issues and its contents: recommendation of individual management plan, correct inhaler technique. There were no significance difference in the perceptions of patients and physicians in the areas of monitoring of peak expiratory flow, using of symptoms/medication diary and asthma support organization (Table 3).

**Table 3:** Physicians and patients reported perception of asthma education and its contents

Asthma Education	Physician (%) n = 70	Patient (%) n = 150	P values
Agreed 50% of visit time was devoted to education	**38.6**	**18.7**	**0.002**
Keep daily symptom/medication diary	**11.4**	**14.7**	**0.051**
Monitor peak expiratory flow	**18.6**	**18.7**	**0.986**
Develop individual management plan	**64.3**	**33.3**	**<0.001**
Correct Inhaler Technique	**84.3**	**70.7**	**0.030**
Contact patient support organizations	**2.0**	**1.4**	**0.741**

### Awareness and impact of side effects

**Figure 1:**
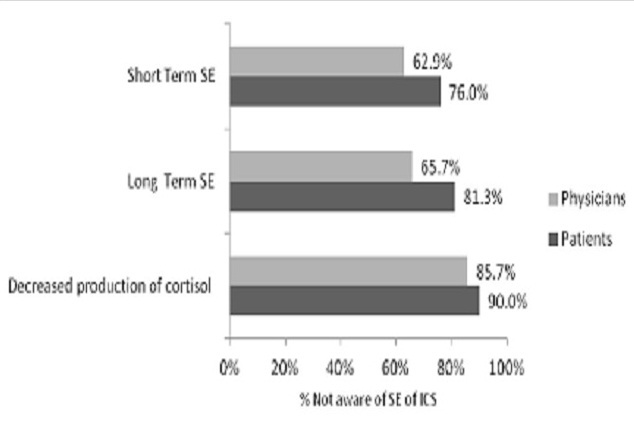
Patients and physicians reported lack of awareness of the side effects of ICS

The majority of patients (60.0%) and physicians (80.0%), p≤0.004 reported that they had discussions with their doctors and patients respectively on medication side effects. Most physicians (61.4%) reported that they initiated discussions in contrast to 29.3% reported by the patients p≤0.001. The patients and physicians reported high percentages of lack of awareness of short term and long term side effects (Figure 1). Only 10.7% of patients have experienced the short term effects of inhaled corticosteroids (ICS) while 20.0% experienced its long term effects. When the patients were asked about the step they took when they experienced medication side effects, 33.3% of them considered switching medications, 29.3% changed the dosage, and 28.0% considered skipping doses, skipped doses and stopped medications respectively. The common reasons for switching medications were reduction of symptoms (38.7%), concern about potential side effects (28.0%), experienced side effects (26.7%) and high cost of medications (21.3%).

### Treatment compliance and symptoms

**Figure 2:**
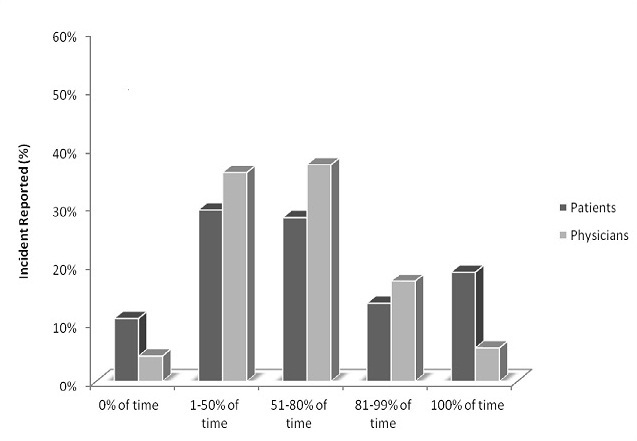
Patient-reported compliance and physician-perceived patient compliance

**Figure 3:**
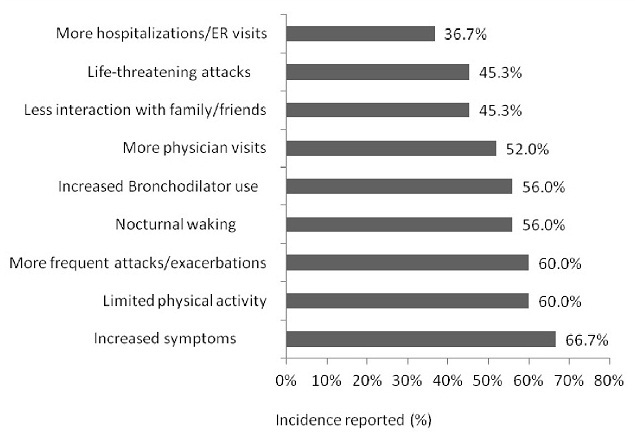
The effect of treatment non-compliance 100% of the time in patients

The patients were questioned about the percentage of the time they took their asthma medication according to their doctor's or healthcare professional's instructions, 18.7% reported that they were 100% compliant and 13.3% were >80% compliant. The physicians were asked the same question and 5.7% of them reported their patients were 100% of time compliant and 17.1% were >80% compliant (Figure 2). Patients who failed to take their asthma medication 100% of time as instructed by doctor or other health care professional’ reported poor quality of life and increase health resource utilization (Figure 3).

### Prescription practices and GINA guidelines

**Figure 4:**
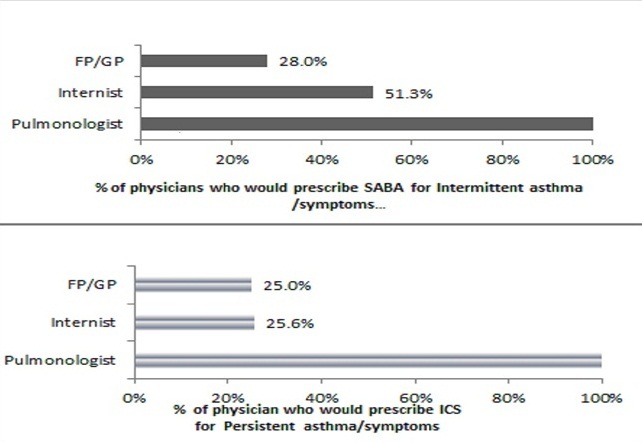
Prescription practices of physician and compliance with GINA Guidelines

We found that most of the participating physicians (81.9%) agreed that Inhaled corticosteroids (ICS) are the “gold standard” treatment for asthma. When the physicians were asked which medication or medications would they prescribe as first-line treatment for adult patients with intermittent and persistent asthma. For intermittent asthma, 100% of pulmonologist, 25.6% of internist, 25.0% of GP/FP would prescribed only a short acting ß 2 agonist SABA, and for persistent asthma all the pulmonologist, half (51.3%) of internist and 28.0% of the GP/FP) would prescribed ICS (Figure 4). About half of the internist (46.2%) and 56.0% of GP/FP admitted that they were ignorant of drugs used as first-line treatment for persistent asthma.

### Perceptions on the need for improved asthma treatment options

**Figure 5:**
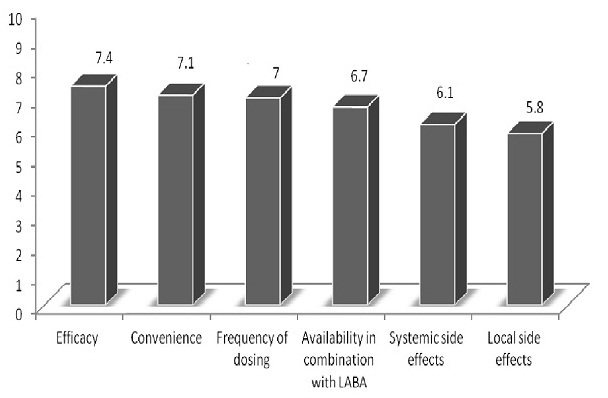
Physician rating of satisfaction of attributes of ICS

The physician respondents were least satisfied with the systemic and local side effect of ICS. The local side effects were greatly rated as a source of great concern and dissatisfaction. Majority of the physicians (85.7%) believed there were unmet needs in the area of ICS therapy, and 56.0% of patients thought there was a need for new medication options for asthma treatment. Asthma patients who described their disease as severe, limited by daily activities and switched medication because of side effects, cost and not satisfied with current medication were more likely to desire for new treatment options (Table 4). Physicians are least satisfied with the availability and side effects of ICS (Figure 5).

**Table 4:** The determinants of the desire for new medications

Determinant	Crude r	Adjusted r	P values
**Impact of asthma & symptom control**			
Asthma severity	+0.39	+0.41	0.001
Limitation of activity	+0.35	+0.34	0.007
Unscheduled visit to your doctor	+0.24	+0.15	0.252
**Awareness & impact of side effects**			
Experienced side effect	+0.34	+0.33	0.008
**Switched /discontinue medication**			
potential side effects	+0.25	+0.19	0.139
expensive medication	+0.26	+0.27	0.033
Inconvenient to use	+0.26	+0.14	0.281
**Satisfaction with the current medication**			
Not satisfied about potential side effects	+0.35	+0.31	0.013

## Discussion

The results of our study shows that the perception of physicians and patients of the educational content of discussion significantly differed in recommendation of individual management plan, correct inhaler technique, discussion of side effects and medication compliance. The level of medication compliance was very low and patients who were not compliant with medications all of the time experienced negative outcomes and increase in resource utilization. The level of reported short term and long term side effects of ICS was low and patients had to skip doses and stopped medications due to side effects. Most physicians (85.7%) and half of patients agreed on the need for new medication options. The need for new medication was strongly related to asthma severity, limitation by daily activities, side effects, cost and lack of satisfaction with current medication. With the exception of pulmonologists, other categories of physicians did not readily prescribed ICS and most of their prescriptions were not in line with treatment guidelines.

In this study, asthma had a serious impact on the patients as 66.7% of them described their asthma as moderate to severe and 62.7% reported a limitation of activity due to the disease and almost half of the patient made unscheduled visits to their doctor or visited the emergency room for asthma attack. This is an indication of lack of disease control in a significant proportion and this finding is in keeping with the GAPP and other previous studies in Nigeria [13–16]. The high level of uncontrolled asthma is an indication of low quality of asthma care in the country which responsibility primarily rest on the physicians, partly on the patients and the healthcare system.

This study has exposed the low level of asthma education in Nigeria as corroborated by both the physicians and patients as one third of the physicians and one fifth of patients reported that half of the clinic visit was devoted to educational issues. In addition to poor level of education, we also observed a significant difference in their perceptions of the correct inhaler technique and development of an individual management plan by the physician and patients. Good asthma knowledge is well correlated to increase medication adherence, improved quality of asthma management, reduced health care utilization and better health outcomes [17]. The low level of asthma education may be attributed to poor knowledge of asthma management by most physicians who were major providers of care in the country. Asthma management goes beyond treating patient in acute attack, the skill and competence of long term care is lacking in most doctors and this fact need to emphasized and addressed to improve the care. Other long term care are lacking in most doctors and this fact need to emphasized and addressed to improve the care Other reasons are lack of consultation time and asthma educators or nurse with an interest in asthma especially when the physicians are running very busy clinics [13, 18]. Lack of support group may also have contributed to low level of asthma education as they are known to offer additional patient support and reinforcement of key educational messages which are very important to overall satisfaction and outcome [19].

More than 70% of the patients were unaware of short and long term side effects of ICS and that physician tend to underestimate the lack of awareness among the patients. This result is in contrast to GAPP study where one third (31%) of patients were unaware of long term side effects [13]. In this same GAPP study, the physicians equally underestimated the lack of awareness of the side effects among patients [13]. Our data also showed that less than one in four experienced the side effects of ICS and this may be attributed to lack of awareness and ability to recognize the side effects. GAPP and other studies found a strong correlation between side effects and the levels of treatment compliance [13, 20, 21].

With regards to treatment compliance, we found that about one in five patients (18.7%) in this study were less likely to be 100% of time compliant with medication, similarly patients in the UK are less likely to comply with treatment instructions, in contrast to 36% in France, 49% of patients in Europe and 48% globally[13]. Patient who failed to take their asthma medications 100% of time as instructed by their doctors’ or other health care professionals’ reported increased morbidity like more symptoms, increased bronchodilator use, increased exacerbations and use of health care facilities. The health care provider can increase treatment compliance among the patients by improving their knowledge of asthma medication, ensuring adequate interaction with them to correct some of the myth and belief such as addiction to asthma medication and prescribing inhaled medication for long term therapy to reduce side effect associated with oral medication.

This study also found that most physicians believed inhaled corticosteroids (ICS) are the “gold standard” treatment for asthma. Inhaled corticosteroids are essential for achieving these goals and managing patients with persistent asthma over the long-term [1, 2]. With regards to knowledge of first-line treatment of persistent asthma, all participating pulmonologists were aware of this line of treatment while, 46.2% of internists and 56.0% of GP/FP admitted their ignorance of the as first-line treatment for persistent asthma or symptoms. From this result, we can infer that ICS is not readily prescribed by FP/GPs and internists and most of the prescriptions were not in line with GINA guidelines. Similar observations have been highlighted in other studies in which most health care providers do not often prescribe inhaled corticosteroids for asthma [1, 13, 22, 23]. The low prescription of ICS in our study may be due to the lack of knowledge of guidelines by physicians.

Majority of physicians and patients believed there were unmet needs in the area of ICS therapy and there is a need for new medication options for treatment of bronchial asthma. Our result is in support of the need for new therapeutic options to meet the patients’ expectations and ensure compliance. The demand for new therapy may be explained by multiple dosages per day as reported by the patients in this study, lack of satisfaction with the use of ICS due to in correct inhalation device and subsequent ineffective delivery of the medication to the airway. This lack of satisfaction has made many patients to show preference for the oral medication despite the physician recommendation of inhaled medication. Another reason for request for a new therapy is the erroneous belief that that ICS does not appear to significantly modify the course of the disease and are not curative, because asthma symptoms and inflammation rapidly recur when the treatment is discontinued and this is a cause of concern as many patients who are also afraid of being addicted to the medications [24]. The side effects of ICS are also causes for concern among the physicians in this study. The safety of long-acting beta-agonists (LABA) in the treatment of asthma has been a source of concern [25]; however recent meta-analysis has shown that when it is administered concomitantly with ICS mortality is drastically reduced. [26]. ICS that meet the demands of both physician and patients will improve medication compliance and rate of physician prescription.

The strength of this study is that it was conducted in five out of the six geographical region of Nigeria which is a good representation of country. It also included private hospitals that render primary care service and derive their patronage mainly from the higher socioeconomic class. The private hospitals and its clientele have often been excluded in previous asthma studies. The study is however limited by the few numbers of patients from inaccessible and remote rural areas who may have more serious challenges than those in sub- urban and urban areas. The non validation of the questionnaire in native languages used in about 4% of patients as Nigeria has over 200 local languages.

## Conclusion

In conclusion, this study has highlighted poor medication compliance which is related to side effects, lack of patients’ physician communication, poor prescription practices and lack of satisfaction with current medication as potential barriers to asthma treatment. These barriers often lead to poor asthma management and high prevalence of uncontrolled asthma, increased health resources utilization and cost of management in a large cohort of patients [27]. The correction of this communication gap will provide the asthma patients with needed information; skills and training so that they can self control the disease and adjust treatment according to a medication plan developed with the health care provider. Studies have shown that effective communication is associated with good adherence and positive impact on the health outcomes [28–29]. The physician continuing professional development on asthma needs to emphasize patient-focused care and promote good prescription practices. With the huge investment and intense effort by drug companies, a discovery of novel classes of therapy for asthma may be in sight. We need to address these treatment gaps and provide new medication options to improve the quality of asthma care in Nigeria.
